# Foliar nitrogen and phosphorus stoichiometry of three wetland plants distributed along an elevation gradient in Dongting Lake, China

**DOI:** 10.1038/s41598-017-03126-9

**Published:** 2017-06-06

**Authors:** Feng Li, Han Gao, Lianlian Zhu, Yonghong Xie, Guishan Yang, Cong Hu, Xinsheng Chen, Zhengmiao Deng

**Affiliations:** 1 Key Laboratory of Agro-ecological Processes in Subtropical Region, The Chinese Academy of Sciences, Changsha, Hunan 410125 China; 2 Dongting Lake Station for Wetland Ecosystem Research, Institute of Subtropical Agriculture, Changsha, 410125 China; 3 Key Laboratory of Watershed Geographic Sciences, Nanjing Institute of Geography and Limnology, Chinese Academy of Sciences, Nanjing, 210008 China; 4 College of Education, Hunan Agricultural University, Changsha, Hunan 410128 China

## Abstract

We examined foliar nitrogen (N) and phosphorus (P) stoichiometry of 3 wetland plants (*Phalaris arundinacea*, *Miscanthus sacchariflorus*, and *Carex brevicuspis*) distributed along an elevation gradient in the Dongting Lake, China, and how this stoichiometry is related to soil physico-chemical characteristics, elevation, and flooding days. Plant and soil samples were collected from 3 lakeshore sites. Total N and P concentrations of plants and six physico-chemical characteristics of the soil were measured, in addition to the elevation and flooding days. *P. arundinacea* and *M. sacchariflorus* had higher total N and P concentrations than *C. brevicuspis*. The foliar N:P ratio decreased with increasing elevation, and only increased with increasing foliar total N concentration. Canonical correspondence analysis indicated that the foliar stoichiometry was primarily regulated by soil water content, followed by soil nutrient concentration. The foliar N and P stoichiometry of the 3 wetland plants was insignificantly correlated with soil total P concentration. However, foliar stoichiometric characteristics and soil total N concentration significantly differed among the 3 species. These results demonstrate that spatial variation of foliar stoichiometry in wetland plants exists along an elevation gradient, with this information being useful for the conservation and management of wetland plants in this lake.

## Introduction

Ecological stoichiometry helps to enhance our understanding of the relationship between elemental composition and the growth of organisms from the molecular to global level^1–3^. It provides a way to investigate the ecological interactions of organisms with the surrounding environment^2, 4^. Among plant nutrients, nitrogen (N) and phosphorus (P) are important for the formation of various fundamental compounds^3, 5, 6^. The quantity and ratio of these 2 elements in plants serve as effective indicators of the nutrient limitation and utilisation efficiency of plants^7–9^. Many studies have confirmed that low (<14) and high (>16) foliar N:P ratios indicate N and P limitation, respectively, with transitional states occurring at N:P ratios of between 14 and 16^9, 10^.

Changes in the availability of N and P lead to changes in plant traits, vegetation composition, and species diversity^11, 12^. In some North American wetlands, the structure of plant communities changes significantly with nutrient gradients, while species richness declines with increasing nutrient availability^13^. The Resource Ratio Hypothesis suggests that when the limiting resource in a given vegetation community changes, the dominant species changes due to altered growth performance and competition. Thus, the ecological stoichiometry of plants is an important indicator of the distribution patterns of plants^9, 14^. To date, ecological stoichiometry has been widely used as an effective indicator on whether species are able to coexist, along with food-web dynamics and nutrient cycling^10, 12^; however, few studies have analysed the relationship between plant distribution and ecological stoichiometry. Moreover, existing studies have primarily focused on forest ecosystems^9, 12, 15, 16^, with studies on wetlands remaining limited.

Plant zonation along environmental gradients is a common phenomenon in wetlands^17, 18^. Such zonation exists in Dongting Lake, China, which is the second largest freshwater lake and the most typical river-connected lake in China, due to its large water exchange capacity with the Yangtze River. Dominant plant communities in this lake are distributed along an elevation gradient: high-elevation species, such as *Miscanthus sacchariflorus* and *Phragmites australis*, mid-elevation species, such as *Carex brevicuspis* and *Polygonum hydropiper*, and low-elevation species, such as *Phalaris arundinacea*. To date, most studies have focused on the mechanism that leads to this pattern in distribution^18, 19^. However, the relationship between plant ecological stoichiometry and plant distribution has not yet been clarified.

In the present study, we focused on the foliar stoichiometric characteristics (including total N and P concentrations and N:P ratio) of 3 plant species (*P. arundinacea*, *C. brevicuspis*, and *M. sacchariflorus*) along an elevation gradient in Dongting Lake. The soil physico-chemical characteristics in these 3 plant communities (including pH, conductivity, soil water content, and total nitrogen, phosphorus, and organic carbon concentrations) were also analysed, as well as elevation and the number of days of flooding (termed flooding days). Specifically, we aimed to: (1) examine the foliar stoichiometric characteristics of these 3 plant species distributed at different elevations, and (2) examine the relationships between plant foliar stoichiometric characteristics and soil physico-chemical characteristics, elevation, and flooding days.

## Materials and Methods

### Study site and plants

**Figure 1:**
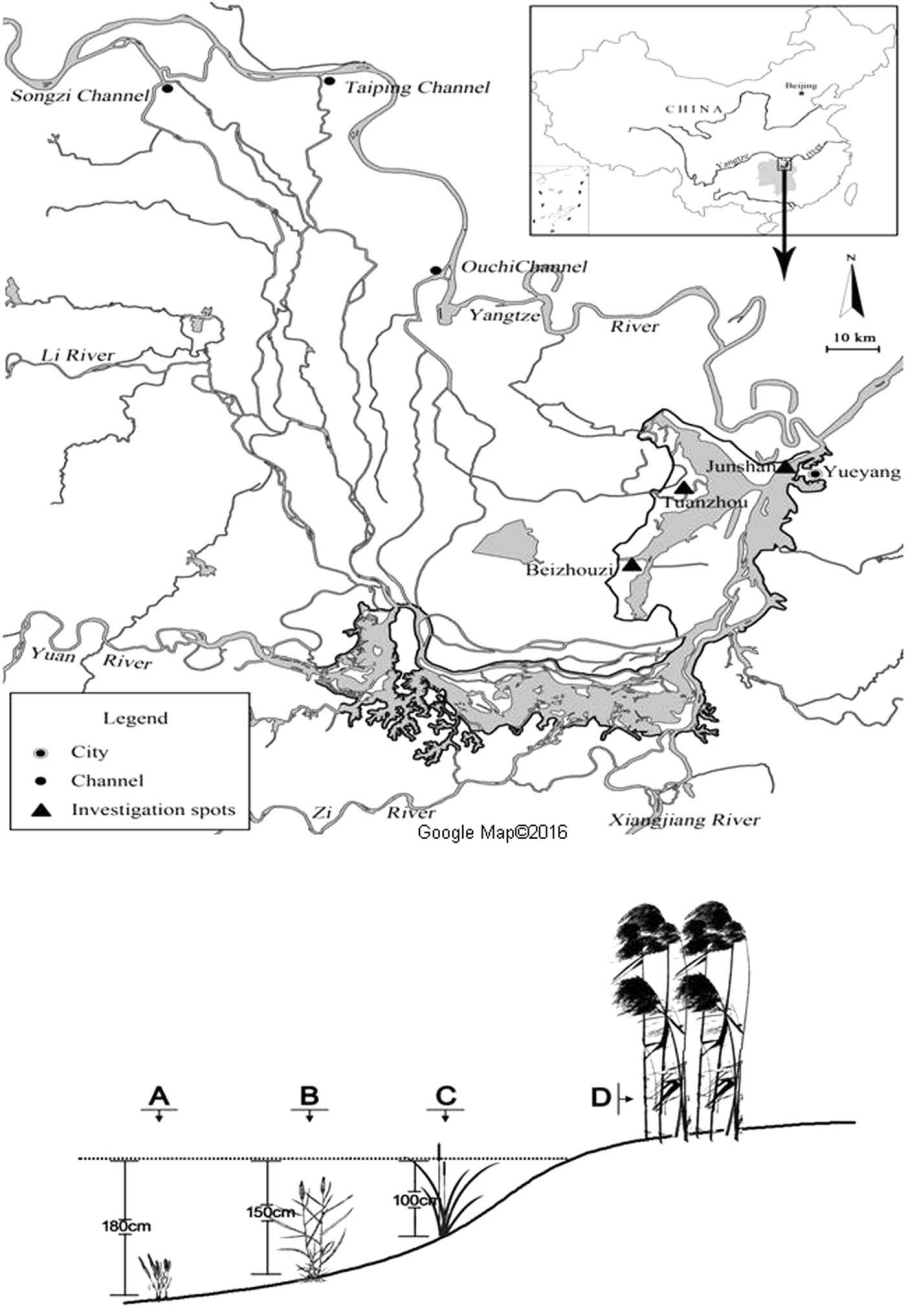
A: Water system and location of sampling sites in Dongting Lake. The shaded areas show the wetlands. B: Distribution patterns of common plants along a water level gradient in Dongting Lake: (**A**) submerged macrophytes, (**B**) *Phalaris arundinacea*, (**C**) *Carex brevicuspis*, and (**D**) *Miscanthus sacchariflorus* (Fig. 1A was generated with software Photoshop 7.0).

Dongting Lake (28° 30′–30° 20′ N, 111° 40′–113° 10′ E) is located on the south bank of the middle reach of the Yangtze River, which receives inflow from four rivers (Xiang, Zi, Yuan, and Li) in Hunan Province and four channels (Songzikou, Taipingkou, Ouchikou, and Tiaoxiankou) connected to the Yangtze River (Fig. 1). The wetlands are characterised by large seasonal fluctuations in water level, and are usually completely flooded from May to October, while being susceptible to drought from November to April. The mean annual temperature is 16.8 °C, with hot summers (June to August, 27.3 °C) and cold winters (December to February, 5.8 °C). The mean annual precipitation is 1,382 mm, with more than 60% of rain falling in April to August.

The present study was conducted in 3 lakeshore areas of Dongting Lake: Chapanzhou (28° 54′ 11.5″ N, 112° 48′ 34.6″ E), Beizhouzi (29° 09′ 22.7″ N, 112° 47′ 18.4″ E), and Junshan (29° 24′ 18.4″ N, 113° 04′ 35.7″ E; Fig. 1). Three dominant plant species were studied in these lakeshore areas, which represented the dominant species, exhibiting a pattern of zonation along an elevation gradient. *P. arundinacea* is a perennial plant with a rugged stem reaching a height of about 60–150 cm. *C. brevicuspis* is a perennial acaulescent herb reaching a height of 40–110 cm. *M. sacchariflorus* is a perennial herb with an erect culm that grows 4–5 m height, with a diameter of 1.5–1.8 cm.

### Field surveys

In May 2015 (i.e. before the onset of flooding), sampling sites were established in the lakeshore areas containing the 3 plant communities (*M. sacchariflorus*, *C. brevicuspis*, and *P. arundinacea*). At each lakeshore area, a belt transect of about 1 km length was established in the middle of each community. Then, 6 quadrats (1 × 1 m) were established at 100 m intervals along each belt transect. The coordinates of each quadrat were recorded using a global positioning system (UniStrong, MG758E). Plant density, above ground biomass (fresh weight), height, and coverage were recorded. Plant density was defined as the number of plants in each quadrat. Plant height was defined as the length of the plant from the ground to the top leaf, and was measured 3 times in each quadrat using a 0.1-cm steel tape (Table 1). Then, the mature leaves were collected from plants in each plot. Leaves of similar size, shape, and location (middle of each plant) were selected from plants of similar height. Then, all above-ground parts was collected from each transect and taken back to the Key Laboratory of Agro-ecological Processes in the Subtropical Region, Chinese Academy of Sciences, where they were oven-dried at 70 °C for 72 h, and the mass was measured using an electronic scale with 0.01 kg precision.

**Table 1:** Community characteristics of wetland plants (means ± SE) distributed along an elevation gradient of 3 lakeshore areas in Dongting Lake, China.

Location	Species	Height (cm)	Coverage (%)	Density(plant m^2^)	Biomass (g m^2^)
Chapanzhou	*P. arundinacea*	124.3 ± 5.5b	83.3 ± 0.0b	358.1 ± 56.1b	209.0 ± 26.9a
	*C. brevicuspis*	102.3 ± 2.5a	97.1 ± 0.0c	786.7 ± 0.1c	288.0 ± 24.6a
	*M. sacchariflorus*	295.0 ± 3.4c	58.3 ± 0.0a	21.3 ± 1.8a	684.8 ± 53.5b
Beizhouzi	*P. arundinacea*	128.3 ± 4.7a	78.3 ± 0.0b	184.0 ± 14.9b	205.3 ± 22.7a
	*C. brevicuspis*	108.5 ± 3.6a	97.8 ± 0.0c	874.7 ± 42.3c	354.0 ± 37.7b
	*M. sacchariflorus*	303.6 ± 11.9b	65.0 ± 0.0a	21.0 ± 0.7a	541.8 ± 42.7c
Junshan	*P. arundinacea*	127.8 ± 3.8b	95.0 ± 0.0c	504.0 ± 84.2b	448.8 ± 34.5ab
	*C. brevicuspis*	96.7 ± 2.4a	86.8 ± 0.0b	880.0 ± 37.4c	408.0 ± 35.6a
	*M. sacchariflorus*	295.0 ± 15.7c	64.2 ± 0.0a	41.6 ± 8.3a	550.6 ± 30.5b

Different letters indicate significant differences among treatments at the 0.05 significance level.

After the plants were surveyed and sampled, soil samples were collected. In each quadrat, five 0–20 cm depth soil cores were collected; specifically, 1 central and 4 corner cores. The samples were then mixed thoroughly into 1 composite sample. The soil samples were placed in polyethylene bags and transported to the laboratory, where they were kept at 4 °C until analysis. The samples were processed within 20 days.

In addition, the elevation of each quadrat was calculated from its coordinates and using a digital elevation model (1:10,000) of Dongting Lake created in 1995 (Changjiang Water Resources Commission, Ministry of Water Resources, China), with an accuracy of 0.1 m. The flooding days of each quadrat were calculated based on elevation and daily water level data (08:00) obtained from the Chenglingji Hydrological Gauging Station during 2014 (Table 2).

**Table 2:** Physico-chemical characteristics (means ± SE) of soil in the 3 wetland plant communities distributed along an elevation gradient of 3 lakeshores areas in Dongting Lake, China.

Location	Species	pH	Conductivity (us cm^−1^)	Water content (%)	Total nitrogen concentration (mg g^−1^)	Total phosphorus concentration (mg g^−1^)	Organic carbon (mg g^−1^)	Elevation (m)	Flooding days
Chapanzhou	*P. arundinacea*	8.1 ± 0.1b	278.2 ± 10.7	38.6 ± 1.1c	0.9 ± 0.1a	0.8 ± 0.0a	11.6 ± 0.3a	25.0 ± 0.1b	179.0 ± 0.8b
	*C. brevicuspis*	7.9 ± 0.0ab	289.8 ± 11.7	30.8 ± 1.8b	2.0 ± 0.2b	0.9 ± 0.0b	17.4 ± 1.4b	24.3 ± 0.1a	187.2 ± 1.6c
	*M. sacchariflorus*	7.8 ± 0.1a	281.8 ± 62.7	22.8 ± 1.0a	1.3 ± 0.1a	0.8 ± 0.0a	16.1 ± 1.6b	28.5 ± 0.0c	127.2 ± 0.2a
Beizhouzi	*P. arundinacea*	7.9 ± 0.0	294.8 ± 27.5b	37.8 ± 3.0b	2.5 ± 0.1b	0.9 ± 0.0a	21.4 ± 1.1b	26.2 ± 0.2a	158.5 ± 4.1b
	*C. brevicuspis*	7.9 ± 0.0	363.9 ± 21.9c	49.0 ± 2.2c	2.8 ± 0.4b	1.0 ± 0.0b	28.8 ± 3.4c	26.3 ± 0.1a	154.0 ± 0.9b
	*M. sacchariflorus*	8.0 ± 0.1	177.6 ± 10.7a	24.6 ± 2.6a	1.0 ± 0.2a	0.9 ± 0.0a	11.6 ± 1.7a	29.4 ± 0.1b	86.0 ± 0.0a
Junshan	*P. arundinacea*	7.9 ± 0.0ab	222.8 ± 5.5a	33.2 ± 1.1c	1.4 ± 0.1a	0.9 ± 0.1b	14.9 ± 0.3	22.1 ± 0.0a	236.0 ± 0.0c
	*C. brevicuspis*	7.8 ± 0.0a	277.3 ± 17.7b	44.4 ± 2.9b	1.9 ± 0.2b	0.7 ± 0.0a	19.0 ± 0.7	22.7 ± 0.1b	224.0 ± 0.5b
	*M. sacchariflorus*	8.0 ± 0.1b	252.8 ± 7.6ab	22.2 ± 1.0a	1.7 ± 0.3ab	0.7 ± 0.0a	17.9 ± 3.2	26.4 ± 0.2c	153.5 ± 1.9a

Different letters indicate significant differences among treatments at the 0.05 significance level.

### Laboratory analysis

All of the leaf samples were oven-dried at 70 °C to a constant weight, and were ground for further analysis. Leaf N concentration was measured with a flow injection analyser (FIAstar 5000, FOSS, Sweden), while leaf P concentration was measured by the molybdenum blue colorimetric method after the leaf samples were digested in an H_2_SO_4_ + H_2_O_2_ solution.

Soil samples were air-dried and sieved to remove coarse fragments (<0.5 mm for organic carbon and total N concentration; <0.1 mm for total P concentration; <2 mm for other analyses). Soil pH was determined from a solution containing a 1:2.5 ratio (w/v) of soil to distilled water using a Mettler Toledo 320 pH meter (Mettler-Toledo Instruments Co., Ltd., China). Soil organic carbon concentration was measured by wet oxidation with KCr_2_O_7_ + H_2_SO_4_, and titrated with FeSO_4_. Total soil N concentration was measured using the Kjeldahl method, and total soil P concentration was determined by acid digestion with an H_2_SO_4_ + HClO_4_ solution. Concentrations were expressed based on oven-dried soil weight. Soil water content was determined by drying soil samples in an oven at 105 °C for at least 72 h.

### Data analysis

A general linear model (GLM), with vegetation community as a fixed factor and sample site as a random factor, was used to analyse whether plant stoichiometric characteristics, including total N and P concentrations, and the N:P ratio differed significantly among the 3 communities. Multiple comparisons of means were performed using Tukey’s test, and a Bonferroni correction for multiple comparisons was applied when necessary. Data were log10-transformed, if necessary, to reduce heterogeneity of variances. Normality and homogeneity were tested using Liljefors’ and Levene’s tests, respectively.

How plant stoichiometric characteristics were correlated with soil properties, elevation, and flooding days were analysed by Canonical Correspondence Analysis (CCA). The vegetation data matrix included plant stoichiometric characteristics (total N and P concentrations, and N:P ratio) of the 3 species. The environmental data matrix consisted of soil properties (pH, conductivity, water content, total N and total P concentrations, organic carbon concentration), elevation, and flooding days. CCA was conducted using CANOCO ver.4.5 (Plant Research International, Wageningen, The Netherlands). Plant stoichiometric characteristics and soil-property ordination diagrams were prepared with CanoDraw LITE to illustrate the results^20^. The relationships between different indexes of plant stoichiometric characteristics and soil total N and total P concentrations were analysed using SPSS 15.0 software. Moreover, we performed curve estimation from which the “best fit” relationship for each statistical analysis was selected, i.e. the highest R^2^ and the lowest P value.

## Results

### Foliar N and P concentrations, and N:P ratio

**Figure 2:**
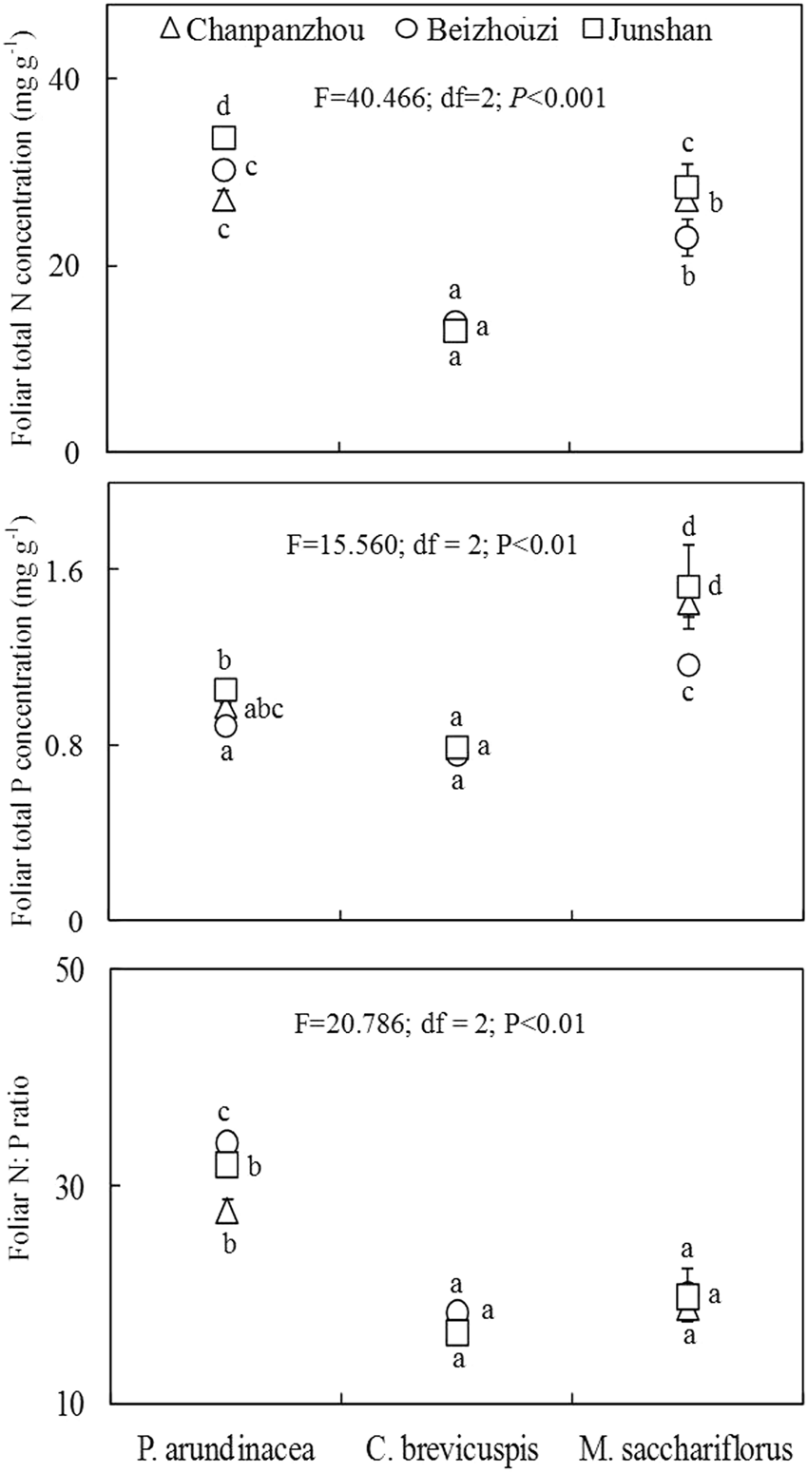
Foliar total nitrogen (N) and total phosphorus (P) concentrations, and foliar N:P ratio of the 3 wetland species distributed along an elevation gradient in 3 lakeshore areas of Dongting Lake, China.

Foliar N and P concentrations were significantly influenced by vegetation type (Fig. 2). The average total N concentration of *P. arundinacea*, *C. brevicuspis*, and *M. sacchariflorus* in the 3 lakeshore areas was 30.45 mg g^−1^, 13.48 mg g^−1^, and 26.26 mg g^−1^, respectively. The highest foliar total N concentration was detected in *P. arundinacea*, which was 2.0–2.6 times higher than the lowest foliar total N concentration in *C. brevicuspis*. The average total P concentrations of *P. arundinacea*, *C. brevicuspis*, and *M. sacchariflorus* in the 3 lakeshore areas was 0.98 mg g^−1^, 0.78 mg g^−1^, and 1.38 mg g^−1^, respectively. *M. sacchariflorus* had the highest foliar total P concentration, which was 1.5–1.9 times higher than the lowest foliar total P concentration in *C. brevicuspis*. The foliar N:P ratio of *P. arundinacea* (27.86–34.08) was much higher than that in the other 2 species. In contrast, *C. brevicuspis* (16.60–18.45) had a similar N:P ratio to *M. sacchariflorus* (18.95–20.11) (Fig. 2).

**Figure 3:**
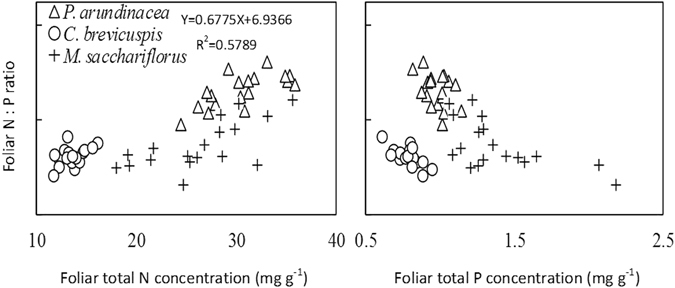
Relationship of foliar N:P with foliar total nitrogen (N) and total phosphorus (P) concentrations of the 3 wetland species distributed along an elevation gradient in 3 lakeshore areas of Dongting Lake, China.

The foliar N:P ratio showed a linear relationship with plant total N concentration; specifically, the foliar N:P ratio increased significantly as total N concentration increased (Fig. 3). However, there was no significant correlation between the N:P ratio and total P concentration (Fig. 3).

### Canonical correspondence analysis

**Figure 4:**
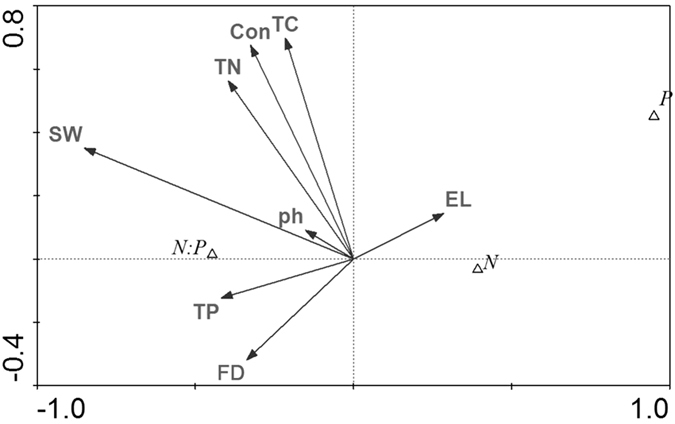
Canonical correspondence analysis (CCA) ordination for the foliar stoichiometric indexes and soil environmental characteristics of the 3 wetland species distributed along an elevation gradient in 3 lakeshore areas of Dongting Lake, China. N: foliar total nitrogen (N) concentration; P: foliar total phosphorus (P) concentration; N:P: ratio of foliar total N and total P concentrations; pH: soil pH; Con: soil conductivity; TC: soil organic carbon concentration; SW: soil water content; TN: soil total nitrogen concentration; TP: soil total phosphorus concentration; EL: elevation; FD: flooding days.

The first and second axes of the CCA ordination explained approximately 97.4 and 99.7% of total variance of the species-environment relationship, respectively (Table 3; Fig. 4). The first axis was negatively correlated with soil water content, total N concentration, and total P concentration. The second axis was positively correlated with soil organic carbon concentration and conductivity.

**Table 3:** Summary of Canonical Correspondence Analysis (CCA) ordinations.

Environmental factors	Axis 1	Axis 2
Soil pH	−0.1214	0.0335
Soil conductivity	−0.2603	0.2487
Soil water content	−**0.6814**	0.1292
Soil total N concentration	−**0.3170**	0.2073
Soil total P concentration	−**0.3346**	−0.1588
Soil organic C concentration	−0.1721	0.2573
Elevation	0.2285	0.0530
Flooding days	−0.2700	−0.1175
Eigenvalues	0.010	0.000
Species-environment correlations	0.801	0.369
Cumulative percentage variance of species data (%)	57.0	58.5
Cumulative percentage variance of species-environment relation data (%)	97.4	99.7

### Relationship between foliar stoichiometric characteristics and soil total N and total P concentrations

**Figure 5:**
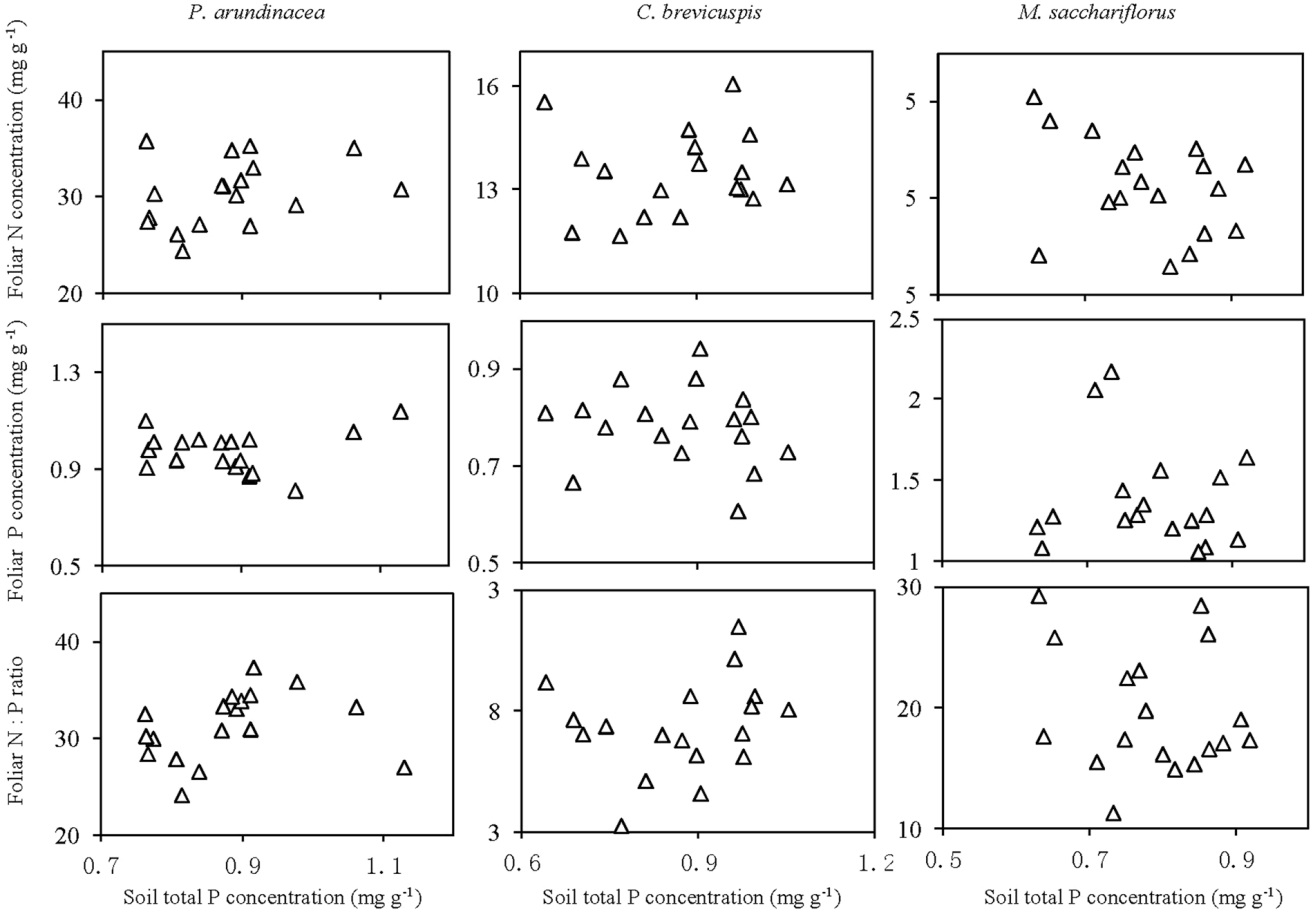
Relationship of foliar stoichiometric indexes with soil phosphorus (P) of the 3 wetland species distributed along an elevation gradient in 3 lakeshore areas of Dongting Lake, China.

**Figure 6:**
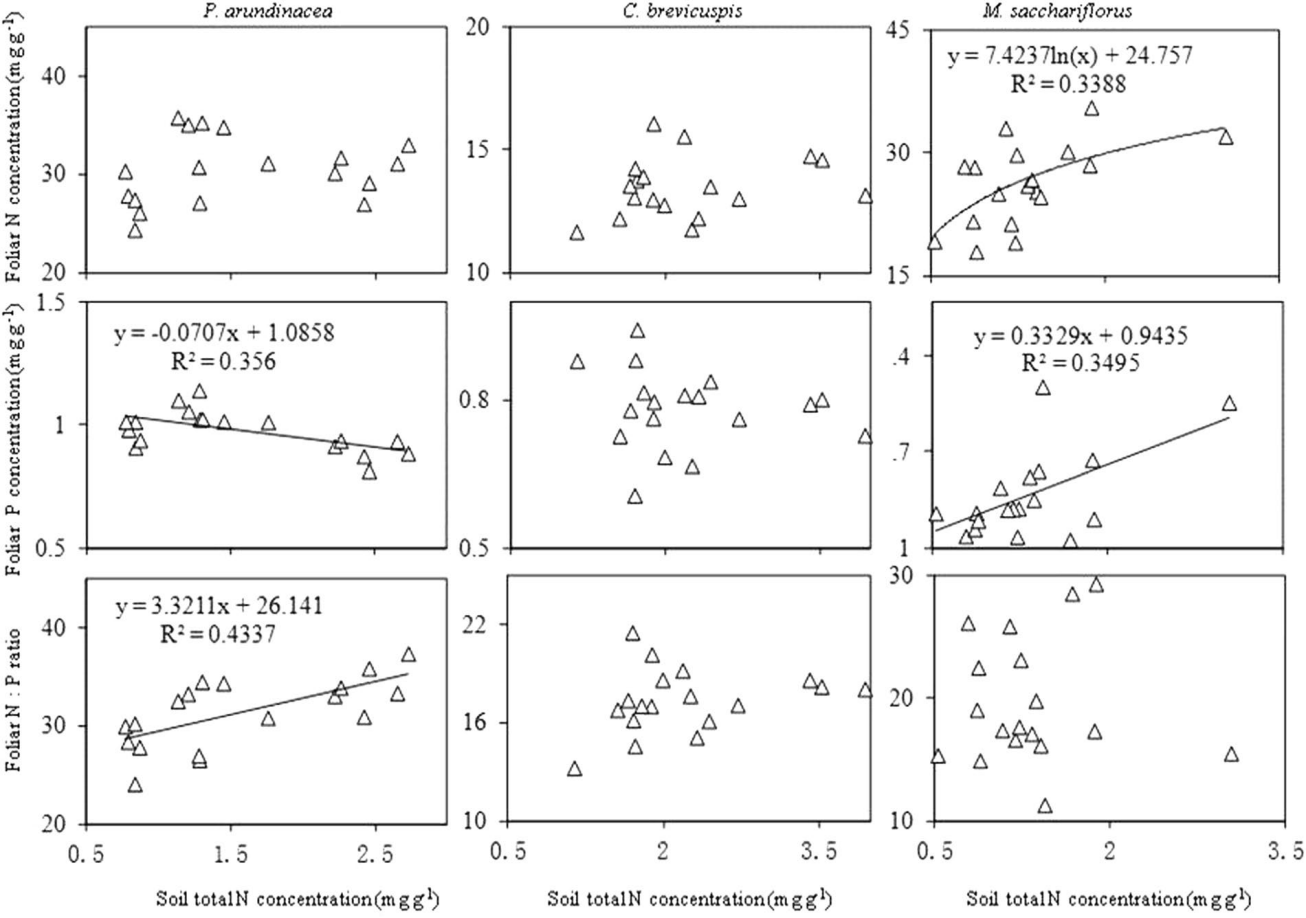
Relationship of foliar stoichiometric indexes with soil total nitrogen (N) of the 3 wetland species distributed along an elevation gradient in 3 lakeshore areas of Dongting Lake, China.

For all the 3 species, foliar total N, total P, and N: P ratio showed no significant relationship with soil total P concentration (Fig. 5). The relationship between foliar stoichiometric characteristics and soil total N concentration varied significantly among the 3 species (Fig. 6). For *P. arundinacea*, foliar P concentration and the N:P ratio showed a negative linear correlation. In comparison, the foliar P concentration of *P. arundinacea* showed a positive linear correlation with soil total N concentration. For *C. brevicuspis*, no significant relationship was detected between foliar stoichiometric characteristics and soil total N concentration. For *M. sacchariflorus*, foliar N concentration and P concentration showed a logarithmic correlation, while foliar N and soil N concentration showed a positive linear correlation (Fig. 6).

## Discussion

The total N and P concentrations of the 3 studied species were consistent with the results of previous studies on aquatic macrophytes in wetlands in China. In eastern China, N and P concentrations of aquatic macrophytes were 6.5–59.4 mg g^−1^ and 0.6–8.6 mg g^−1^, respectively^3^. A study of 52 wetland sites across China calculated N and P concentrations of 1.8–45.1 mg g^−1^ and 0.1–9.9 mg g^−1^, respectively, in aquatic macrophytes^21^. Furthermore, our results confirmed that total N and total P concentrations, as well as the N:P ratio, differed significantly among the 3 studied macrophyte species. This variation might be due to foliar elemental concentrations and ratios being primarily determined by the genetic and physiological characteristics of plants, rather than external environmental factors^22, 23^. Demars and Edwards also showed that the variance in the plant tissue nutrient concentrations of 378 species in the River Sprey (northeast Scotland, UK) catchment could be explained by species identity^24^. In the current study, the N:P ratio was highest in *P. arundinacea*, which was distributed at the lowest elevation. This ration was mostly due to the higher N concentration and lower P concentration in this species. Because *P. arundinacea* occupies a lower elevation compared to the other 2 species, it is inundated for longer. Anaerobic conditions produced by long-term inundation increase the solubility and mobilization of iron from soils^25^. This phenomenon coupled with regular flushing, might explain the lower P concentration and higher N:P ratio in *P. arundinacea*.

Soil nutrients have a strong influence on plant growth and distribution, as well as being the primary source determining the concentration of nutrients in plants^6, 9^. The present study showed that the highest soil nutrient concentration (including total N, total P, and organic C concentrations) occurred in the *C. brevicuspis*, rather than in the other 2 species. Plant litter decomposition, flooding, and sedimentation all contribute towards determining soil nutrient concentrations in this lake. For instance, the sedimentation rate is usually higher in the *P. arundinacea* community compared to the other 2 communities, due to it occurring at a lower elevation. As a result, nutrient concentrations in the *P. arundinacea* community are likely to be higher. However, the soil of the *P. arundinacea* community is more frequently flooded than the other 2 communities, because of it being distributed at a lower elevation. Thus, the litter of *P. arundinacea* could be easily washed away by flooding before full decomposition, leading to significantly lower soil nutrient levels. Moreover, in this lake, *M. sacchariflorus* is the main raw material used for papermaking, with an annual harvest. Consequently, the nutrients of this species were not being returned to the soil, which might explain the low soil nutrient concentrations in this community.

The foliar N:P ratio ranged from 16.6 to 34.1 among the 3 studied macrophytes, indicating that growth was P limited based on the criterion (P limitation when N:P > 16) proposed by Koerselman and Meuleman^26^. The obtained foliar N:P values were higher than the mean foliar N:P ratio at the global scale (11.8), and might be caused by low P levels^27, 28^. The P concentration of the 3 macrophytes in the current study was 25.4–50.9% of the emergent plants in the wetlands of eastern China^3^. Han *et al*. also reported low foliar P concentrations for wetland vegetation in China compared to global averages^29^. Low P levels might be due to P in the soil being highly insoluble compared to N. Furthermore, a dense root system is usually needed for plants to extract significant amounts of P from soil^13^. However, in Dongting Lake, these 3 macrophyte species tend to have shallow root systems to acclimate to flooding stresses, which might be unfavourable for P absorption. Furthermore, previous studies also found that P is generally deficient in soil of subtropical regions^30^. This phenomenon might also explain the low P concentration in wetland plants in this lake. Moreover, our study confirmed that the foliar N:P ratio increased with increasing total N concentration, but had no significant correlation with total P concentration. This result also confirmed that P is the limited nutrient in this wetland, supporting a previous study conducted in the karst ecosystems of southwest China^9^.

Soil water content is important for determining plant distribution and nutrient absorption, especially in freshwater wetlands^18^. Our CCA results confirmed that soil water content was the primary factor influencing the ecological stoichiometry of plants in Dongting Lake. Higher soil water content increased anaerobic conditions, reduced rhizosphere microbial activity, and influenced many biochemical processes (e.g. nitrification, N mineralisation), which subsequently limited the ability of plants to extract nutrients^31^. However, this suggestion is based on our preliminary field investigation. Thus, more research is still needed to investigate how soil water content regulates plant stoichiometric characteristics. Moreover, our correlation analysis indicated that the total P concentration of soil had no significant influence on plant stoichiometry. In contrast, the total N content of soil had a different effect on the 3 different species. Variability in the nutrient concentrations of plants might occur because of the life-form, physiological stage of development, nutrient-absorbing ability, and environmental conditions^24^. These parameters might explain the poor relationship between soil nutrients and plant stoichiometry.

In conclusion, our results confirmed that foliar total N and P concentrations differed significantly among the 3 studied macrophytes, with the foliar N:P ratio being higher in *P. arundinacea*, which occurred at lower elevations than the other 2 species. Our study also showed that soil water content is the primary factor influencing the ecological stoichiometric characteristics of wetland macrophytes in this lake. These results provided preliminary insights towards understanding the relationships between plant stoichiometric characteristics and plant distribution patterns, which could be used to enhance the conservation and management of the wetland plants in this lake. In recent years, the water level of Dongting Lake has decreased significantly, due to a combination of global climate change and anthropogenic disturbance (e.g. construction of the Three Gorges Dam). This phenomenon has led to significant changes in plant distribution patterns, as demonstrated by a decrease in the minimum elevation distribution of the 3 studied species^32^. Thus, future studies need to focus on how these change influence plant stoichiometry.
